# Treatment and Diagnostic Challenges in a Patient with Atypical SARS-CoV-2-Associated Encephalitis Mimicking a Neoplasm: A Case Report

**DOI:** 10.3390/reports9030258

**Published:** 2026-08-06

**Authors:** Marios Theologou, Panagiotis Kyriakongonas, Nikolaos Syrmos, Theologos Theologou

**Affiliations:** 1 Department of Neurosurgery, General Hospital of Thessaloniki Georgios Papanikolaou, 57013 Thessaloniki, Greece; 2 First Department of Neurosurgery, IASO General Clinic, 15123 Athens, Greece; 3 Department of Neurosurgery, 401 General Military Hospital of Athens, 11525 Athens, Greece; 4 School of Medicine, Aristotle University of Thessaloniki, 54124 Thessaloniki, Greece

**Keywords:** COVID-19 encephalitis, Long-COVID, glioma mimic, stereotactic biopsy, focal impaired awareness seizures, facial nerve palsy, histopathology

## Abstract

**Background and Clinical Significance:** Encephalitis is a rare neurological complication associated with Severe Acute Respiratory Syndrome Coronavirus 2 (SARS-CoV-2) infection. In rare cases, focal neuroinflammation can manifest as a mass-like parenchymal lesion, creating profound diagnostic and treatment dilemmas by mimicking primary central nervous system neoplasms. **Case Presentation:** A 34-year-old female presented with cephalalgia, nausea, confusion, facial palsy, and a new onset of focal impaired awareness seizures (FIAS). Brain magnetic resonance imaging (MRI) revealed a prominent hyperintense lesion within the left temporal lobe with associated vasogenic edema and focal leptomeningeal enhancement highly suspicious of a low-grade glial neoplasm. Although nasopharyngeal RT-PCT was negative, the presence of serum anti-SARS-CoV-2 IgM and IgG suggested recent subclinical SARS-CoV-2 infection. To resolve diagnostic ambiguity and avoid empiric oncological overtreatment, a stereotactic brain biopsy was performed. Histopathology revealed acute neuroinflammation characterized by reactive gliosis, microglial hyperplasia, and perivascular lymphatic cuffing, with no evidence of neoplastic presence. Quantitative tissue RT-PCR confirmed the presence of SARS-CoV-2 (Ct33). Follow-up imaging demonstrated complete resolution of the abnormalities following conservative treatment with corticosteroids and antiepileptics, though mild clinical symptoms persisted for 12 months thereafter. **Conclusions:** Encephalitis presents a rare yet critical manifestation of SARS-CoV-2. Establishing definitive etiology remains challenging. Stereotactic biopsy is a valuable tool to guide appropriate treatment in cases of ambiguous imaging and clinical findings. Radiographic resolution may precede complete clinical recovery.

## 1. Introduction and Clinical Significance

Encephalitis is a rare inflammatory condition of the brain parenchyma, with high morbidity and mortality, and is associated with infectious agents (e.g., herpes simplex virus-1, and varicella-zoster virus) or autoimmune processes [1,2]. The neurotropism of SARS-CoV-2 is well-documented. Neurological manifestations are driven by direct cellular damage (neuroinvasion) and immune system hyperactivation. They can range from mild (anosmia, ageusia, dizziness, and headache) symptoms to severe, life-threatening conditions (ischemic stroke, encephalitis, seizures, meningitis, cranial polyneuritis, and Guillain–Barré syndrome) [3]. Differentiating encephalitis from intracranial neoplasms can be highly challenging due to similar neuroimaging and clinical findings, risking misdiagnosis and unnecessary oncological treatment. We present a case of atypical SARS-CoV-2-associated encephalitis mimicking a glial tumor and discuss its pathophysiology and diagnostic and treatment challenges, highlighting the necessity for a stereotactic biopsy and the time difference between imaging and clinical recovery.

## 2. Case Presentation

**Figure 1:**
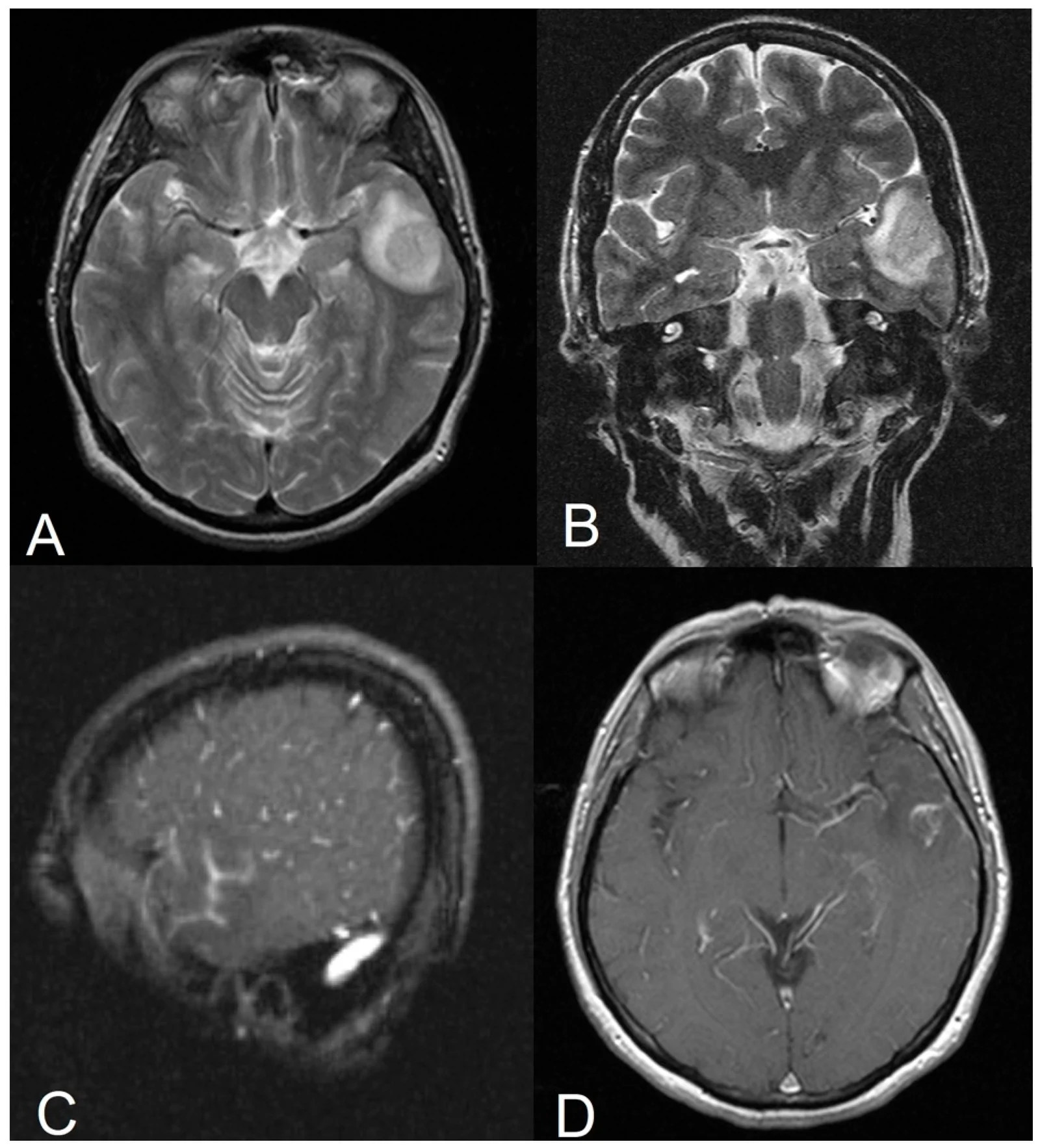
(**A**) Axial and (**B**) coronal T2-weighted MR images presenting abnormal hyperintensity within the anterior aspect of the left temporal lobe. Notable changes include focal gyri thickening, effacement of the adjacent sulci, blurring of the subcortical gray–white matter junction and concomitant predominantly vasogenic edema. (**C**) Sagittal and (**D**) axial T1-weighted post-contrast images revealing prominent focal leptomeningeal enhancement in the left anterior temporal region and adjacent lateral frontal operculum. The radiographic differential diagnosis primarily includes focal encephalitis versus a low-grade glial neoplasm.

A 34-year-old Roma female presented to the emergency department with a new onset of focal impaired awareness seizures (FIASs). Ten days prior, she had experienced gradual neurological deterioration characterized by sudden, severe retro-orbital headaches accompanied by nausea and progressive confusion. The pain was described as stabbing, lasting approximately one hour, recurring multiple times per day at irregular intervals, not responding to standard analgesics, exacerbated by activity and forward bending, and partially relieved when recumbent. Neurological assessment was unremarkable, except for mild right-sided central facial palsy (slight weakness and asymmetry during movement but absent during rest with full eye closure). No clinical signs of meningitis were present. Computerized tomography (CT) scan revealed minor signs of non-specific focal edema in the left temporal lobe, characterized by a subtle area of low attenuation. Brain magnetic resonance imaging (MRI) registered abnormal signal intensity within the left temporal lobe; the differential diagnosis based on neuroimaging included either an acute focal inflammatory process or an underlying low-grade glial neoplasm (Figure 1).

Laboratory evaluation revealed an Erythrocyte Sedimentation Rate (ESR) of 24 mm/h, a C-reactive protein (CRP) level of 7.30 mg/L, a lactate dehydrogenase (LDH) level of 316 U/L, a creatine phosphokinase (CPK) level of 199 U/L and a fibrinogen (FIB) level of 394 mg/dL. D-dimmers were elevated at 1031 μg/dL and profound lymphocytopenia (0.6 K/μL) was observed. All other parameters of basic blood biochemistry were within normal limits. Electroencephalography (EEG) findings were normal. The patient was admitted and pharmaceutical therapy was initiated by introducing Levetiracetam (2000 mg/day in two doses) for seizure prophylaxis and intravenous dexamethasone (16 mg/day in four divided doses) to manage perilesional edema. A diagnostic spinal tap was performed; Cerebro-Spinal Fluid (CSF) cytobiochemical analysis and opening pressure were within normal limits. Respiratory tract samples, blood and CSF FilmArray^®^ testing, conventional assays and cultivations were negative for the presence of pathogens, including mycoplasma pneumoniae.

A detailed retrospective medical history revealed that she manifested self-limiting flu-like symptoms (dry cough, fatigue, sore throat, and myalgia) over a five-day period four weeks prior to the onset of neurological manifestations. Her other prior medical history was unremarkable.

While the nasopharyngeal swab Reverse Transcription Polymerase Chain Reaction (RT-PCR) test was negative for SARS-CoV-2 RNA, a double immunochromatography blood test assay revealed the presence of SARS-CoV-2-specific IgG and IgM immunoglobulins (Igs). Intrathecal SARS-CoV-2 antibodies were not detected on immunochromatography, and the CSF SARS-CoV-2 RT-PCR was negative. Comprehensive autoimmune CSF and serum panels—including assays for autoantibodies against mGluR1, mGluR5, GABA_A_R, DPPX, Iglon5, anti-Hu, anti-Yo, anti-Ri, anti-CV2, anti-amphiphysin, anti-M1, and anti-M2—as well cancer index blood markers, were all negative. Additional serologic immunofluorescent testing for neurotropic viruses was negative except for Cytomegalovirus (CMV) IgG and Herpes Simplex Virus ½ (HSV) IgG, consistent with past exposure. CSF was negative for HHV-6, CMV and HSV1/2, while IgG specific to Varicella Zoster Virus (VZV) and measles were not found in the CSF sample. Epstein–Barr Virus (EBV) and influenza were similarly ruled out via serology and nasopharyngeal RT-PCR, respectively.

She had been fully vaccinated according to the national immunization schedule but had not received any SARS-CoV-2 or influenza vaccines.

A Total-Body Computerized Tomography (CT) was performed, revealing mild emphysematous lesions in the para-diaphragmatic pulmonary/lung region and a few bilateral ground-glass opacifications in the posterior lung regions; however, no other pathological findings were registered.

**Figure 2:**
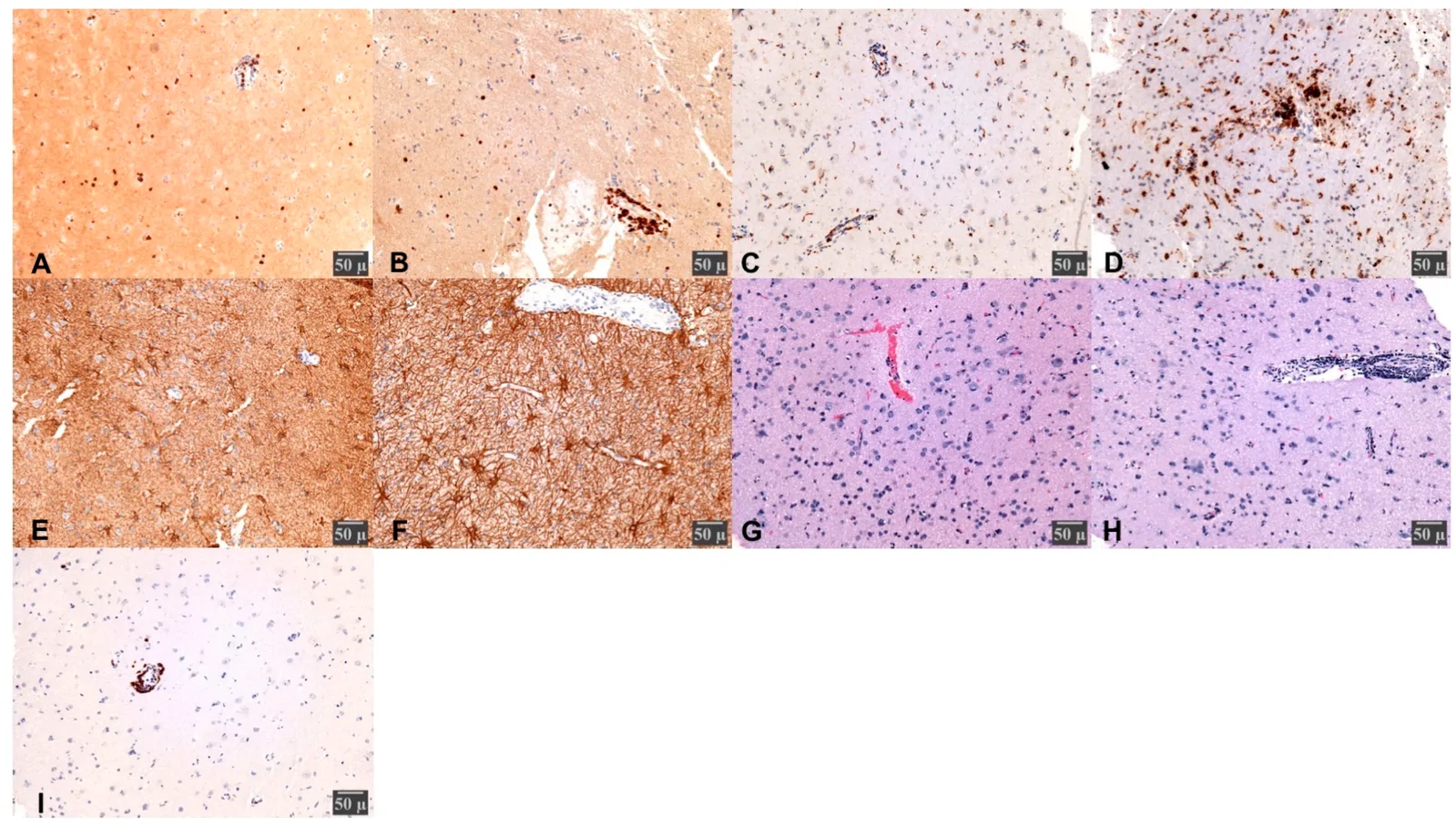
Microscope photography of white (wm) and gray matter (gm) (scale provided). Hematoxyline–Eosine (gm: (**G**) and wm: (**H**)) and immunohistochemical dies [incl. vimentin, GFAP (gm: (**E**) and wm: (**F**)), MAP2, DIA-H09, p53, Ki67-MIB1, CD68, LCA, CD3 (gm: (**A**) and wm: (**B**)), CD6 (gm: (**C**) and wm: (**D**)) and CD20 (wm: (**I**))] were employed. The specimen presented a normal brain architecture with normal cell nuclei. Signs of reactive gliosis are registered (**F**,**G**) alongside perivascular infiltration of macrophages and T/B lymphocytes and microglial hyperplasia (**C**,**D**).

Given the diagnostic ambiguity, the neurosurgical team proceeded with a stereotactic brain biopsy. A total of nine target specimens were harvested from the left temporal region. Histopathology findings were consistent with atypical encephalitis (Figure 2). Viral RNA analysis was performed on 20 μm thick paraffin-embedded sections. The tissue-specific SARS-CoV-2 PCR was positive with a cycle threshold (Ct) of 33.

**Figure 3:**
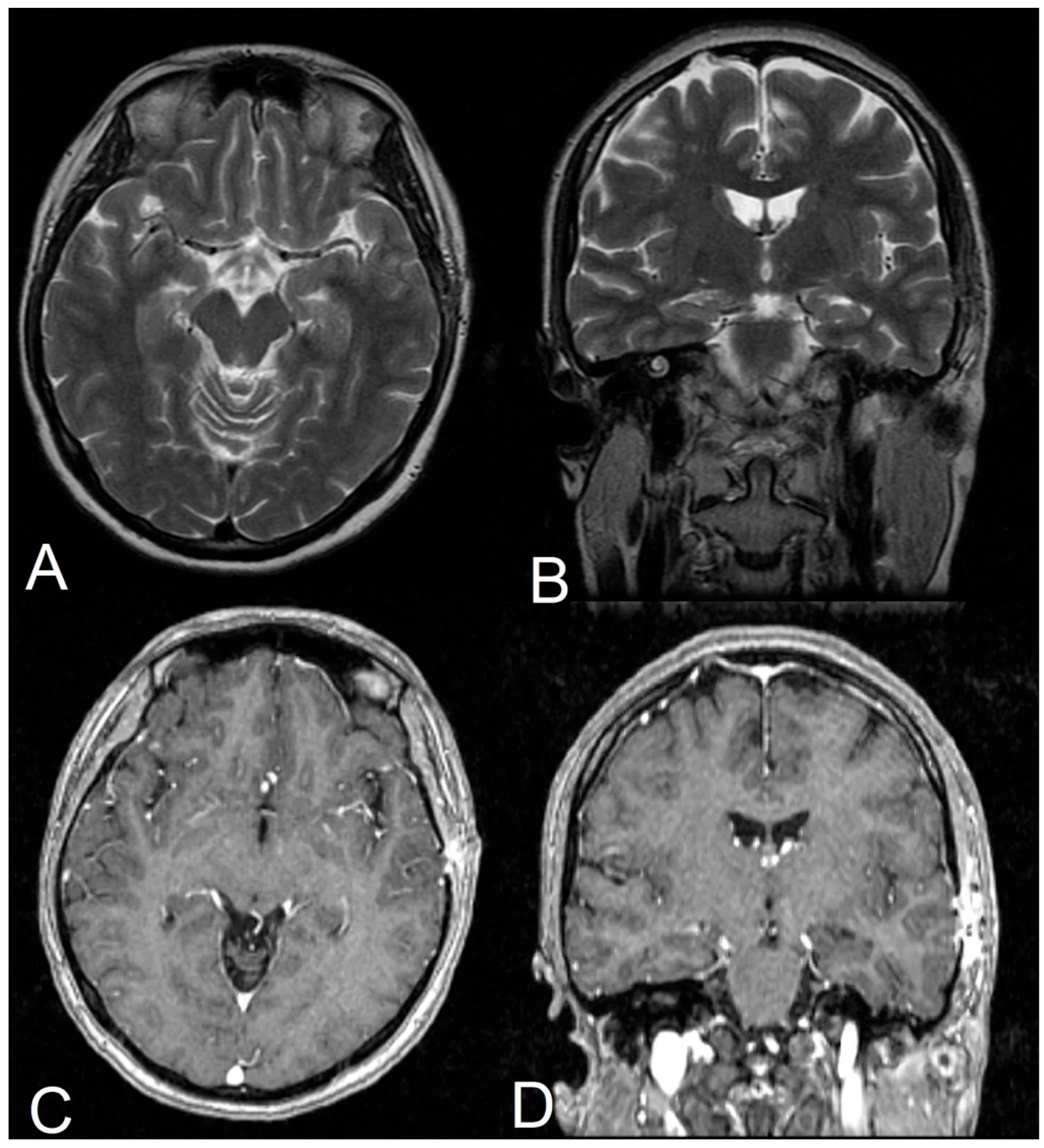
(**A**) Axial and (**B**) coronal T2-weighted images showing resolution of previously described findings. (**C**) Axial and (**D**) coronal T1-weighted post-contrast application showing minor leptomeningeal enhancement.

The patient’s postoperative course was uneventful. She was discharged on a continuing medical treatment regimen (corticosteroids and an antiepileptic) supplemented empirically with oral azithromycin (750 mg/day) for one month. A follow-up MRI obtained three weeks post-discharge revealed near-complete resolution of the previously described findings (Figure 3). Her neurological deficit normalized within a month. However, the patient’s severe cephalalgia accompanied by a reported difficulty in concentrating and thinking persisted and gradually improved over a six-month period. Due to depressive mood swings and short-term memory deficits, the patient was referred for supportive neuropsychiatric therapy, ultimately achieving full functional recovery one year later. Levetiracetam was discontinued after 12 months, with the patient being seizure-free for 24 months before being lost to follow-up.

## 3. Discussion

The neurotropism of SARS-CoV-2 occurs via hematogenous dissemination mediated by angiotensin-converting enzyme 2 (ACE-2) receptors or via retrograde axonal transport along the olfactory, glossopharyngeal and vagus nerves using ACE-2, neuropilin 1 (NRP1) and transmembrane serine protease 2 (TMPRSS2) [4,5,6,7,8,9]. Direct viral invasion and the secondary inflammatory immune response cause microvascular dysregulation and disruption of the blood–brain barrier (BBB), resulting in vasogenic edema. Symptoms are typically mild (cephalalgia, dizziness, ageusia, olfactory and visual impairments); however, in rare cases, the clinical presentation can be severe (delirium, encephalopathy/encephalitis, ataxia, seizures, increased risk of stroke, and hyperactivation of the sympathetic system) [10,11,12,13,14,15].

Differentiating acute encephalitis from central nervous system (CNS) neoplasms in neuroimaging may be challenging [16,17], especially in patients without non-neurologic clinical symptoms and signs of infection. The presented patient had only neurologic symptoms upon admission, retrospectively recalling self-limiting flu-like symptoms.

While neurological manifestations of SARS-CoV-2 are predominantly associated with severe infections [18], a subset of patients can exhibit them following mild or asymptomatic cases [13,14,15].

The clinical course of our patient explains the initial diagnostic ambiguity. The screening nasopharyngeal RT-PCR swab at admission was negative, yet serological assays confirmed the presence of serum SARS-CoV-2 IgM and IgG antibodies, correlating with prior flu-like symptoms. While rapid immunochromatography is not recommended for acute infection screening (as immunoglobulins require time to reach a detectable concentration), it serves as a valuable diagnostic tool for patients presenting with delayed neurological manifestations after the resolution of respiratory symptoms or in asymptomatic individuals [19,20,21]. A negative nasopharyngeal swab cannot exclude a prior or localized CNS infection, as the virus may potentially clear from the upper respiratory tract while remaining latent or contained within other deeper tissues, including neural structures such as the trigeminal ganglion [4,22]. In virology, classical latency refers to a clinically dormant phase where the virus ceases replication while its genome remains within host cells, with minimal expression and the potential for reactivation. Although SARS-CoV-2 has not shown a potential of reactivation in humans, its RNA or proteins can persist for many weeks or even months in different tissues, a phenomenon associated with post-acute sequelae of SARS-CoV-2 infection (PASC) or Long-COVID [23,24]. According to the World Health Organization (WHO), PASC is characterized by a wide range of symptoms (most commonly fatigue, myalgia, arthralgia, headache, concentration impairment and taste loss) with an onset within three months of the initial infection and lasting a minimum of two months. However, this patient presented with distinct neurological symptoms and signs and an objective MRI-documented lesion, extending beyond standard PASC criteria.

Magnetic resonance imaging (MRI) remains the gold standard for evaluating these lesions. While initial computed tomography (CT) imaging demonstrated minor signs of non-specific focal edema, subsequent MRI scans revealed a well-demarcated lesion localized in the left temporal lobe with associated leptomeningeal enhancement. This matches the topographical predilection of SARS-CoV-2 for the temporal lobes and limbic structures described in the literature [16,25,26].

Clinically, our patient presented a mild course, in contrast with the majority of reported cases, which frequently required admission to the intensive care unit (ICU) due to refractory epileptic seizures [27]. The transient right central facial nerve palsy observed here suggests potential brainstem involvement, confirming the strong predilection proposed in the literature [4], though it should be highlighted that no direct association was established with imaging or laboratory data.

Confirming a viral etiology in the CNS remains challenging. In line with previous reports, the patient’s CSF analysis was cyto-biochemically normal, and herCSF RT-PCR was negative for SARS-CoV-2. This is common, as viral shedding into the subarachnoid space is often transient, and CSF viral titers can remain below detectable thresholds even during active parenchymal inflammation [28,29,30].

To avoid empiric oncological overtreatment, a stereotactic brain biopsy was performed. In a similar case, the authors proceeded directly to an anterior temporal lobectomy under the assumption that the lesion was a glioma, discovering post-viral encephalitis only upon histopathological examination [16]. While their intervention successfully controlled the reported refractory seizures, our surgical team advocates for a stereotactic biopsy whenever neuroimaging cannot definitively differentiate malignancy from an inflammatory process. Our patient’s histopathology confirmed acute neuroinflammation—characterized by reactive gliosis, perivascular lymphocytic cuffing, macrophage infiltration, and microglial hyperplasia—without neoplastic cells, which matched a positive quantitative tissue RT-PCR result.

Currently, there is no single, universally established protocol for SARS-CoV-2 encephalitis. Most reported cases have been managed empirically with a combination of antimicrobial and immunomodulator agents. Dexamethasone was utilized in our patient to mitigate at least the perilesional edema, a reasonable approach [31] despite ongoing debate regarding the benefits of its use in treating encephalitis [32,33]. Azithromycin was administered empirically in accordance with the prevailing practice at that time due to its hypothesized immunomodulatory, anti-inflammatory and neuroprotective properties [34]. We should emphasize that the recent literature defies potential benefits; thus, its use is not advisable [35].

This case highlights that complete clinical neuropsychiatric recovery may lag behind the normalization of neuroimaging and laboratory markers. The persistence of post-infectious headaches, memory impairment, and depressive symptoms underscores the necessity of a multidisciplinary follow-up protocol. Patients recovering from focal neuroinflammation should be routinely screened for long-term cognitive and psychiatric sequelae.

## 4. Conclusions

SARS-CoV-2-associated encephalitis can present as a localized, mass-like temporal lobe lesion mimicking a neoplasm. In cases of diagnostic ambiguity, a negative nasopharyngeal RT-PCR result cannot exclude the disease; a detailed retrospective clinical history and serum immunoglobulin testing are essential to uncover recent subclinical and biphasic infections. When a definite diagnosis cannot be confidently placed non-invasively, a stereotactic brain biopsy with tissue-specific RT-PCR should be performed prior to definitive surgical intervention to prevent overtreatment. Neuroimaging resolution may precede complete cognitive and neuropsychiatric recovery, highlighting the necessity for a long-term multidisciplinary follow-up plan.

## Data Availability

The original contributions presented in this study are included in the article. Further inquiries can be directed to the corresponding author.

## Abbreviations

The following abbreviations are used in this manuscript:

ACE -2	Angiotensin-Converting Enzyme 2
BBB	Blood–Brain Barrier
CMV	Cytomegalovirus
CNS	Central Nervous System
CPK	Creatine Phosphokinase
CSF	Cerebrospinal Fluid
CT	Computed Tomography
Ct	Cycle threshold (used in quantitative PCR analysis)
EBV	Epstein–Barr Virus
EEG	Electroencephalography
ESR	Erythrocyte Sedimentation Rate
FIAS	Focal Impaired Awareness Seizures
FIB	Fibrinogen
GFAP	Glial Fibrillary Acidic Protein
HHV-6	Human Herpesvirus 6
HSV	Herpes Simplex Virus
ICU	Intensive Care Unit
Ig	Immunoglobulin (IgM/IgG)
LDH	Lactate Dehydrogenase
MRI	Magnetic Resonance Imaging
NRP1	Neuropilin-1
PASC	Post-Acute Sequelae of SARS-CoV-2 Infection (Long-COVID)
PCR	Polymerase Chain Reaction
RNA	Ribonucleic Acid
RT-PCR	Reverse Transcription Polymerase Chain Reaction
SARS-CoV-2	Severe Acute Respiratory Distress Syndrome Coronavirus 2
TMPRSS2	Transmembrane Serine Protease 2
VZV	Varicella Zoster Virus
WHO	World Health Organization
